# Hydroxychloroquine's Early Impact on Cone Density

**DOI:** 10.1155/2021/1389805

**Published:** 2021-09-06

**Authors:** Tomoko Ueda-Consolvo, Toshihiko Oiwake, Shinya Abe, Tomoko Nakamura, Ayaka Numata, Atsushi Hayashi

**Affiliations:** Department of Ophthalmology, Graduate School of Medicine and Pharmaceutical Sciences, University of Toyama, Toyama, Japan

## Abstract

**Purpose:**

To evaluate early effects of hydroxychloroquine (HCQ) on the retina using adaptive optics (AO).

**Methods:**

This was a prospective observational single-center study of 29 eyes of 29 patients who had been treated with HCQ for the first time and followed with AO for a minimum of two years. Cone counting was performed in 4 quadrants, nasal, temporal, superior, and inferior, at 0.75 mm from the foveal center. The changes of cone density on AO, visual acuity, and foveal thickness within two years of use were analyzed. The changes of mean cone density of patients whose cumulative dose was over 200 g in 2 years were also assessed. We evaluated the correlation between cone density and cumulative dose of HCQ.

**Results:**

There was no significant decrease in cone density in the first 2 years of HCQ use. VA and foveal thickness did not show obvious change, either. Among 9 patients whose cumulative dose was over 200 g in 2 years, the mean cone density showed no significant change at 6, 12, 18, and 24 months compared with baseline (*P*=0.381, *P*=0.380, *P*=0.281, and *P*=0.534, respectively). There was no correlation between cone density and cumulative dose of HCQ at two years (Spearman's correlation coefficient, *r* = −0.0553, *P*=0.780; *n* = 29).

**Conclusion:**

AO showed no change in cone density in the first two years of HCQ use.

## 1. Introduction

Early effects of hydroxychloroquine (HCQ) on the retina remain largely unexplored. HCQ can effectively treat rheumatologic and dermatologic disorders, particularly in patients with systemic lupus erythematosus (SLE) [1]. HCQ has multiple beneficial effects on survival [2, 3], disease activity [4], and the risk of organ damage [5, 6] and thromboembolic episodes [1, 7, 8]. HCQ toxicity to the retina is, however, not rare among long-term users [9]. The earliest damage is to the photoreceptors, with retinal pigment epithelium (RPE) changes occurring later as the outer nuclear layer degenerates [6, 10, 11]. HCQ concentrates in melanin-containing cells and reduces internal pH of lysosomes, leading to an accumulation of lipofuscin, which may cause the retinal toxicity [12]. HCQ retinopathy (HCQR) is progressive and irreversible, and there is no present therapy. Therefore, patients taking HCQ are recommended to be informed about toxicity risk and proper dose levels and appropriately screened for early detection [13]. The American Academy of Ophthalmology (AAO) guidelines recommend the use of both automated visual field and spectral-domain optical coherence tomography (SD-OCT) as the primary tests [13]. Considering that the primary detectable change is in photoreceptors, the assessment of cone density using adaptive optics (AO) retinal imaging could help detect the definitive signs of toxicity at an early enough stage of HCQR. Debellemaniere et al. reported moderate cone loss in patients with no clinical evidence of maculopathy as HCQ cumulative doses increased [14]. These are relatively uneasy results for patients and medical doctors because it is possible that cone loss starts soon after HCQ is administered. The changes of cone density within a few years of use are still unclear. In this prospective observational single-center study, we examined the cone structure in patients who had been treated with HCQ for the first time and followed for at least 2 years.

## 2. Methods

### 2.1. Patients

We carried out a two-year prospective observational study from November 2015 to October 2018 at a single center (Toyama University Hospital, Japan) in 29 eyes of 29 patients (1 man and 28 women) who were introduced HCQ for the first time. The study was approved by the Institutional Review Board of the University of Toyama, and the procedures used conformed to the tenets of the Declaration of Helsinki. Inclusion criteria were (1) patients with no history of taking HCQ and (2) no advanced cataract, corneal opacity, or vitreous hemorrhage which could interfere with the use of AO fundus imaging. If the quality of AO images was the same in both eyes, the right eye was selected. The exclusion criteria were (1) other retinal diseases such as cone-rod dystrophy, cone dystrophy, retinal inflammatory diseases, autoimmune paraneoplastic retinopathy, or drug toxicity and (2) patients with poor image quality. In total, AO fundus images were taken in 33 patients, and 4 patients were excluded because of the poor quality. All patients underwent comprehensive ophthalmic examinations including measurement of best-corrected visual acuity, measurement of intraocular pressure, and examination by slit-lamp biomicroscopy, OCT (RS-3000 Advance; NIDEK Co., Ltd., Aichi, Japan), Humphrey Field Analyzer (HFA; Carl Zeiss Meditec, Dublin, CA) with the 10-2 Swedish Interactive Threshold Algorithm (SITA) standard program, and rtx1™ AO fundus camera (Imagine Eyes, Orsay, France).

### 2.2. Analysis of Adaptive Optics Images

For each eye, a high-resolution image of the foveal center and 4 more images of the area (nasal, temporal, superior, and inferior) around it were captured by moving the fixation point. i2k Retina software (DualAlign^TM^ LLC, Clifton Park, NY) was used to obtain image alignment and multi-image mosaics. After image processing, we had pictures of an 8° × 8° area of the retina with the fovea in the center. Measurements of cone density were performed automatically using AO Detect Mosaic V2.0b17 (Imagine Eyes, Orsay, France). The axial length (AL) of the eye was required to measure cone density. AL was measured with the OA-2000 (Tomey, Nagoya, Japan) optical axial length biometer. Cone counting was performed in each of the 4 quadrants (nasal, temporal, superior, and inferior) at 0.75 mm from the foveal center. The size of the counting area was chosen to avoid retinal capillaries and set as 80 *μ*m × 80 *μ*m square (software default size). The same measured area was ensured by the location and the shape of retinal capillaries. Changes in the mean cone density at the same locations in each eye were followed up at the baseline and 6, 12, 18, and 24 months after the baseline.

### 2.3. Foveal Thickness Measurement

Foveal thickness was manually segmented and defined as the distance from the vitreoretinal interface to the inner border of the RPE. The observer measured the choroidal thickness using the caliper function built into the linear measuring tool. We determined the choroidal thickness by averaging a horizontal and vertical scan passing through the foveal center.

### 2.4. Statistical Analysis

All statistical analyses were carried out using JMP statistical discovery software (version 14.2.0; SAS Institute, Cary, NC). The paired *t*-test was performed for the comparison of two groups. Spearman's correlation procedure was applied to investigate correlations between cone density and cumulative dose of HCQ at 24 months. Statistical significance was defined as *P* < 0.05.

## 3. Results

Clinical characteristics and HCQ dosage information of the patients are presented in Table 1. Of the 29 patients, 28 were women. The mean age was 43.2 ± 10.9 years. Twenty-eight patients were treated for SLE, and one patient was treated for dermatomyositis. The mean daily dose was 228 ± 44.7 mg. The cumulative dose was 183 ± 56.6 g, on average, and the mean daily dose-to-real body weight ratio was 4.15 ± 0.83 mg/kg. In 9 patients, the cumulative dose was over 200 g in 2 years (Table 2). Mean daily dose-to-ideal body weight (IBW) was 4.06 ± 0.73 mg/kg, and there were no patients at this ratio >6.5 mg/kg.

There was no correlation between cone density and cumulative dose of HCQ at 24 months (*r* = −0.0553, *p* = 0.780).

**Figure 1:**
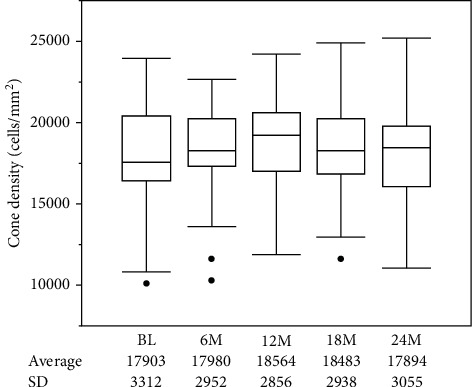
The mean cone density showed no significant change at 6, 12, 18, and 24 months compared with baseline (*P*=0.145, *P*=0.171, *P*=0.097, and *P*=0.866, respectively) (*n* = 29).

**Figure 2:**
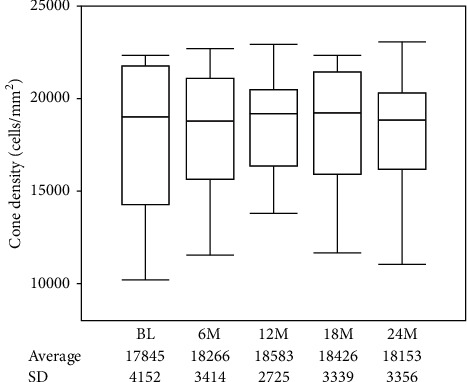
Among 9 patients whose cumulative dose was over 200 g in 2 years, the mean cone density showed no significant change at 6, 12, 18, and 24 months compared with baseline (*P*=0.381, *P*=0.380, *P*=0.281, and *P*=0.534, respectively) (*n* = 9).

The mean cone density showed no significant change at 6, 12, 18, and 24 months compared with baseline (*P*=0.145, *P*=0.171, *P*=0.0973, and *P*=0.866, respectively) (Figure 1). Among 9 patients whose cumulative dose was over 200 g in 2 years, the mean cone density showed no significant change at 6, 12, 18, and 24 months compared with baseline (*P*=0.381, *P*=0.380, *P*=0.281, and *P*=0.534, respectively) (Figure 2 and Table 2). The mean foveal thickness at baseline and 6, 12, 18, and 24 months was 199.2 ± 11.1, 199.1 ± 11.0, 198.8 ± 11.5, 199.8 ± 11.0, and 198.2 ± 11.0, respectively (mean ± standard deviation (SD)). There was no significant change in the mean foveal thickness at 6, 12, 18, and 24 months compared with baseline (*P*=0.842, *P*=0.853, *P*=0.261, and *P*=0.375, respectively). BCVA at baseline and 6, 12, 18, and 24 months was −0.131 ± 0.061, −0.133 ± 0.048, −0.136 ± 0.048, −0.133 ± 0.053, and −0.137 ± 0.053, respectively (mean ± SD). BCVA showed no significant change at 6, 12, 18, and 24 months compared with baseline (*P*=0.842, *P*=0.617, *P*=0.756, and *P*=0.574, respectively).

## 4. Discussion

A novel finding in our study was that there was no significant decrease in cone density in the first 2 years of HCQ use. VA and foveal thickness did not show obvious change, either. To the best of our knowledge, this is the first prospective study on the analysis of the cone density in the initial stage of HCQ introduction using AO.

Stepien et al. found irregularities in the cone density in areas with normal Humphrey visual field (HVF) 10-2 and SD-OCT findings [15]. This finding indicated the potential use of AO as an essential tool for detecting the preclinical stage of HCQ toxicity. Debellemaniere et al. also reported that cone loss may occur at an early stage after exposure to HCQ without clinically evident toxicity. They showed a significant negative correlation between parafoveal cone density and cumulative HCQ dose (*r*^2^ = 0.23, *p* = 0.0018) in patients with no clinical evidence of maculopathy. In 18 of 23 patients, the cumulative dose was over 200 g [14]. In our study, however, there was no correlation between cone density and cumulative dose of HCQ at 24 months (*r* = −0.0553, *P*=0.780). This might be due to the smaller number of patients whose cumulative dose was over 200 g (9 of 29 patients). The negative correlation in Debellemaniere et al.'s study may, as the authors indicate, be the result of the inclusion of both eyes [15]. AO may provide us with valuable information on the natural history of cone survival exposed to HCQ. Since retinal degeneration from HCQ can continue to progress even after the drug is discontinued, detecting early retinopathy is essential. HCQ cessation, however, handicaps patients with autoimmune disease because alternatives to HCQ are more expensive and have more side effects [16, 17]. We need to balance managing autoimmune disease and minimizing the risk of HCQR. Adding assessment of cone loss to the standard clinical test (e.g., fundus photograph, HVF, and SD-OCT) may enable us to monitor HCQR more sensitively. Precise monitoring helps both screening ophthalmologists and prescribing physicians not only for HCQ cessation but also for raising the possibility of reducing daily dosing before drug cessation. It also allows for balancing controlling a systemic disease with protecting patients' vision.

There are potential limitations in this study such as relatively small sample size and gender bias as most of our patients were females. The locations where AO measures with enough reliability and repeatability are still limited. In the current study, cone density was measured in 4 quadrants (nasal, temporal, superior, and inferior) at 0.75 mm from the foveal center. We chose these locations for the following reasons: first, we wanted to accurately and repeatedly measure cone density in the same area over a 2-year span—retinal vessels served as a guide. Second, the region near the fovea is more closely linked to visual acuity than the peripheral areas of the retina. Some limitations were also intrinsic to detecting cone loss because it is still difficult for AO to distinguish cone damage from the artifact. Media opacities and dry eye disease leading to tear film instability and keratitis cause poor image quality. Technical improvements are still needed to acquire AO images in patients on a HCQ regimen. A longitudinal follow-up to the current study is needed to obtain useful information on the long-term cone survival exposed to HCQ.

## 5. Conclusions

AO showed no change in cone density in the first two years of HCQ use. Using AO with standard clinical tests may give us valuable information to calibrate a good balance between controlling autoimmune disease and preserving visual function in patients exposed to HCQ. Long-term studies are needed to examine the follow-up changes in the cone structure and to classify the disease stages of the progressive retinal degeneration in HCQR eyes.

## Figures and Tables

**Table 1:** Demographic data and clinical characteristics of the patients.

Males : females (%)	1 : 28 (3.4 : 96.6)
Mean ± age, y	43.2 ± 10.9
Diagnosis of SLE : dermatomyositis (%)	28 : 1 (96.6 : 3.4)
Daily dose, mg	228 ± 44.7
Body mass index, kg/m^2^	21.9 ± 3.63
Ideal body weight, kg	56.1 ± 4.26
Daily dose/body weight, mg/kg	4.15 ± 0.83
Daily dose/ideal body weight, mg/kg	4.06 ± 0.73
Cumulative dose, g	183 ± 56.6
Cumulative dose/body weight, g/kg	8.51 ± 2.80

SLE: systemic lupus erythematosus.

**Table 2:** Patient demographics and adaptive optics findings.

Patient number	Gender (M/F)	Age (years)	Cumulative dose in 2 years (g)	Pathology	Mean cone density (cells/mm^2^)				
					Baseline	6 months	12 months	18 months	24 months
1	F	46	339.6	SLE	19008 + 3229	18765 + 3870 (*P*=0.711)	19181 + 3481 (*P*=0.702)	18021 + 2696 (*P*=0.064)	18820 ± 3679 (*P*=0.621)
2	F	52	315.7	SLE	17007 ± 2248	18206 ± 2945 (*P*=0.202)	19518 ± 2382 (*P*=0.353)	19953 ± 1981 (*P*=0.290)	18873 ± 2322 (*P*=0.258)
3	F	45	275.1	SLE	22282 ± 2831	22685 ± 3169 (*P*=0.393)	22919 ± 2223 (*P*=0.513)	22320 ± 2399 (*P*=0.947)	23026 ± 1813 (*P*=0.476)
4	M	45	273.2	SLE	10156 ± 5397	13627 ± 3597 (*P*=0.292)	14752 ± 3209 (*P*=0.711)	11629 ± 4668 (*P*=0.471)	11050 ± 444 (*P*=0.860)
5	F	43	227.9	SLE	11507 ± 2761	11561 ± 1006 (*P*=0.974)	13756 ± 1809 (*P*=0.254)	14507 ± 2311 (*P*=0.125)	14082 ± 1580 (*P*=0.180)
6	F	55	226.2	SLE	22300 ± 1893	20691 ± 2924 (*P*=0.222)	18165 ± 2829 (*P*=0.058)	22081 ± 1949 (*P*=0.532)	20531 ± 2711 (*P*=0.248)
7	F	59	222.9	SLE	19566 ± 2145	19694 ± 2998 (*P*=0.823)	19993 ± 2497 (*P*=0.434)	19220 ± 2274 (*P*=0.481)	18709 ± 2122 (*P*=0.164)
8	F	3836	218.4	SLE	17581 ± 2923	17681 ± 2292 (*P*=0.934)	17893 ± 2674 (*P*=0.271)	17266 ± 1622 (*P*=0.866)	18218 ± 1621 (*P*=0.704)
9	F	38	203.2	SLE	21200 ± 2382	21486 ± 1638 (*P*=0.603)	21073 ± 1965 (*P*=0.734)	20840 ± 1058 (*P*=0.678)	20071 ± 869 (*P*=0.299)

M: male; F: female; SLE: systemic lupus erythematosus.

## Data Availability

The data used to support the findings of this study are available from the corresponding author upon request.
